# DNA Methylation Analysis of the Angiotensin Converting Enzyme (ACE) Gene in Major Depression

**DOI:** 10.1371/journal.pone.0040479

**Published:** 2012-07-13

**Authors:** Peter Zill, Thomas C. Baghai, Cornelius Schüle, Christoph Born, Clemens Früstück, Andreas Büttner, Wolfgang Eisenmenger, Gabriella Varallo-Bedarida, Rainer Rupprecht, Hans-Jürgen Möller, Brigitta Bondy

**Affiliations:** 1 Department of Psychiatry and Psychotherapy, Ludwig-Maximilians-University Munich, Munich, Germany; 2 Division of Psychiatric Genetics and Neurochemistry, Ludwig-Maximilians-University Munich, Munich, Germany; 3 Department of Psychiatry and Psychotherapy, University Regensburg, Regensburg, Germany; 4 Institute for Legal Medicine, Ludwig-Maximilians-University Munich, Munich, Germany; 5 Institute for Legal Medicine, University Rostock, Rostock, Germany; 6 Department of Internal Medicine-Preventive Cardiology, Ludwig-Maximilians-University Munich, Munich, Germany; Catholic University of Sacred Heart of Rome, Italy

## Abstract

**Background:**

The angiotensin converting enzyme (ACE) has been repeatedly discussed as susceptibility factor for major depression (MD) and the bi-directional relation between MD and cardiovascular disorders (CVD). In this context, functional polymorphisms of the ACE gene have been linked to depression, to antidepressant treatment response, to ACE serum concentrations, as well as to hypertension, myocardial infarction and CVD risk markers. The mostly investigated ACE Ins/Del polymorphism accounts for ∼40%–50% of the ACE serum concentration variance, the remaining half is probably determined by other genetic, environmental or epigenetic factors, but these are poorly understood.

**Materials and Methods:**

The main aim of the present study was the analysis of the DNA methylation pattern in the regulatory region of the ACE gene in peripheral leukocytes of 81 MD patients and 81 healthy controls.

**Results:**

We detected intensive DNA methylation within a recently described, functional important region of the ACE gene promoter including hypermethylation in depressed patients (p = 0.008) and a significant inverse correlation between the ACE serum concentration and ACE promoter methylation frequency in the total sample (p = 0.02). Furthermore, a significant inverse correlation between the concentrations of the inflammatory CVD risk markers ICAM-1, E-selectin and P-selectin and the degree of ACE promoter methylation in MD patients could be demonstrated (p = 0.01 - 0.04).

**Conclusion:**

The results of the present study suggest that aberrations in ACE promoter DNA methylation may be an underlying cause of MD and probably a common pathogenic factor for the bi-directional relationship between MD and cardiovascular disorders.

## Introduction

Major depression (MD) is one of the most common psychiatric diseases with a lifetime prevalence between 5% and 17% [1]. Although the monoamine hypothesis has been consistently linked to the etiology of MD, the exact pathophysiology remains largely unknown [2]. Actual hypothesis suggest complex interactions between multiple genetic, environmental and epigenetic risk factors that impact the development of MD and severity of symptomatology [3], [4].

Epigenetic processes, mediated through modifications of DNA and histones by non mutagenic mechanisms play a major role in the context of development, but recent studies indicate that epigenetic alterations can also occur due to environmental stimuli during the whole life [5], [6]. One of the most intensely investigated epigenetic mechanisms is the DNA methylation of cytosine residues in regulatory CpG islands of genes. Thus, recent studies have suggested that a dysfunction of DNA methylation in the brain may account for psychiatric disorders, including MD [4], [7], [8].

In this context animal models have shown that early life events in terms of stress, recognized as a major susceptibility factor for depression, can change the DNA methylation level of genes, being implicated in the neurobiology of depression, as glial cell-derived neurotrophic factor (GDNF), glutamic acid decarboxylase 1 (GAD1), estrogen receptor alpha 1b (ERA1B), arginine vasopressin (AVP), glucocrtiocoid receptor (NR3C1) and serotonin transporter (5-HTTP) [9]–[14]. In humans only a few studies of differential DNA methylation patterns in depression have been carried out so far, but the data seem to support those from animal studies. In vitro analysis of lymphoblastoid cell lines of depressed patients revealed an association between MD and increased DNA methylation levels of the 5-HTTP gene [15]. These findings have recently been confirmed in depressed patients [16]. Furthermore, two studies could demonstrate that prenatal exposure to maternal depressed mood decreases the DNA methylation pattern of the methylenetetrahydro-folate reductase (MTHFR) gene and increases the NR3C1 gene methylation in newborn infants [17], [18]. Within a human post mortem study a hypermethylation of the gamma-aminobutyric acid receptor alpha 1 subunit (GABRA1) gene has been reported in the frontal cortex of depressed suicide victims [19]. A genome wide approach to analyze the methylation profile of over 14.000 genes in 33 depressed patients and 67 healthy probands showed coordinated signals with pathophysiological mechanisms previously implicated in the etiology of depression [8]. The findings from these first studies suggest that DNA methylation profiles in several candidate genes may be associated with depression, but the detailed patterns and mechanisms are still unknown.

The angiotensin converting enzyme (ACE), a member of the renin-angiotensin system (RAS) has been repeatedly discussed both as a major candidate gene for depression and as a common susceptibility factor for the bi-directional relation between depression and cardiovascular disorders (CVD) [20]. Cross-sectional and prospective analyses have shown that depression may increase mortality and morbidity in patients with heart failure, regardless of its etiology, and independently of traditional cardiovascular factors. There is now compelling evidence that a reciprocal relationship between both disorders exists. The presence of cardiovascular disease can influence mood states and some of the factors associated with depression, especially the multiple alterations associated with acute and chronic stress, may give rise to vascular disorders such as atherosclerosis, microcirculatory endothelial dysfunction, or metabolic conditions such as diabetes and dyslipidemia [21]. As both disorders are complex and multifactorial in origin, involving multiple genes with interactive or additive effects together with environmental factors, depression and cardiovascular disease could be different manifestations of the same genetic and/or epigentic substrate.

Several genetic studies, especially with the functional ACE Ins/Del polymorphism, from our and other groups propose an involvement of ACE gene polymorphisms in the pathophysiology of major depression, in the therapeutic outcome, in hypothalamic-pituitary-adrenal (HPA)-axis dysregulation and expression of ACE serum concentrations [22]–[27]. The mostly analyzed ACE Ins/Del polymorphism accounts for ∼40%–50% of the ACE serum concentration variance, the remaining half is probably determined by other genetic, environmental or epigenetic factors, but these have not been successfully identified so far.

In this context, within an interdisciplinary study investigating the relation between CVD and MD, we determined the serum concentrations of ACE and several inflammatory biomarkers in depressed patients as probable CVD risk markers. Besides the observation that many inflammatory markers were increased in depressed patients, we observed a significant correlation between the ACE serum concentrations and several of these markers as ICAM-1, VCAM-1, E-selectin, P-selectin and MCP-1 in these patients, independent from the ACE Ins/Del polymorphism (unpublished data).

Based on the above described findings, we hypothesize that epigenetic mechanisms of the ACE gene might be a reason for these observations. Therefore the main aim of the present study was to analyze the DNA methylation in the regulatory region of the ACE gene in peripheral leukocyte DNA of 81 depressed patients compared to 81 healthy controls. The identified methylation pattern was additionally investigated in human post mortem brain tissue (cortex, hippocampus) of 13 control individuals. Furthermore, the DNA methylation pattern was correlated to ACE serum concentrations, to the above described alterations in CVD markers and to clinical characteristics.

## Results

We investigated the DNA methylation pattern in the 5′-region of the ACE gene in peripheral leukocyte DNA from blood of 81 depressed patients and 81 healthy controls. There were no significant differences between patients and controls regarding gender or age (table 1).

**Table 1:** Demographic characteristics of the depressed patients (MD) and healthy controls (CON).

	MD (N = 81)	CON (N = 81)
Sex [males/females]	30/51	40/41
Age [years, mean ± SD];(range)	45.8±14.3 (21–76)	46.2±14.2 (19–73)
Age of onset[years, mean ± SD]	37.2±12.6	
Episodes [mean ± SD]	3.7±2.4	
HAMD-17 [mean ± SD]	22.5±5.5	
ACE-Concentration[Units ± SD]	44.4±15.4	46.2±14.2

### DNA Methylation Levels of the ACE Gene in Patients Controls and Post Mortem Brain Tissue

The CpG island analysis of a 7540 bp region, which contains putative promoter sequences and exon 1, revealed a 1398 bp CpG island (−567 to 831, related to the transcription start site). After bisulphite sequencing intensive methylation was found from nucleotide −456 to −255. In the other parts of the CpG island there was no, respectively very rare, negligible methylation. Thus, the following analysis and evaluations refer to the methylated region from nucleotide −456 to −255.

**Figure 1:**
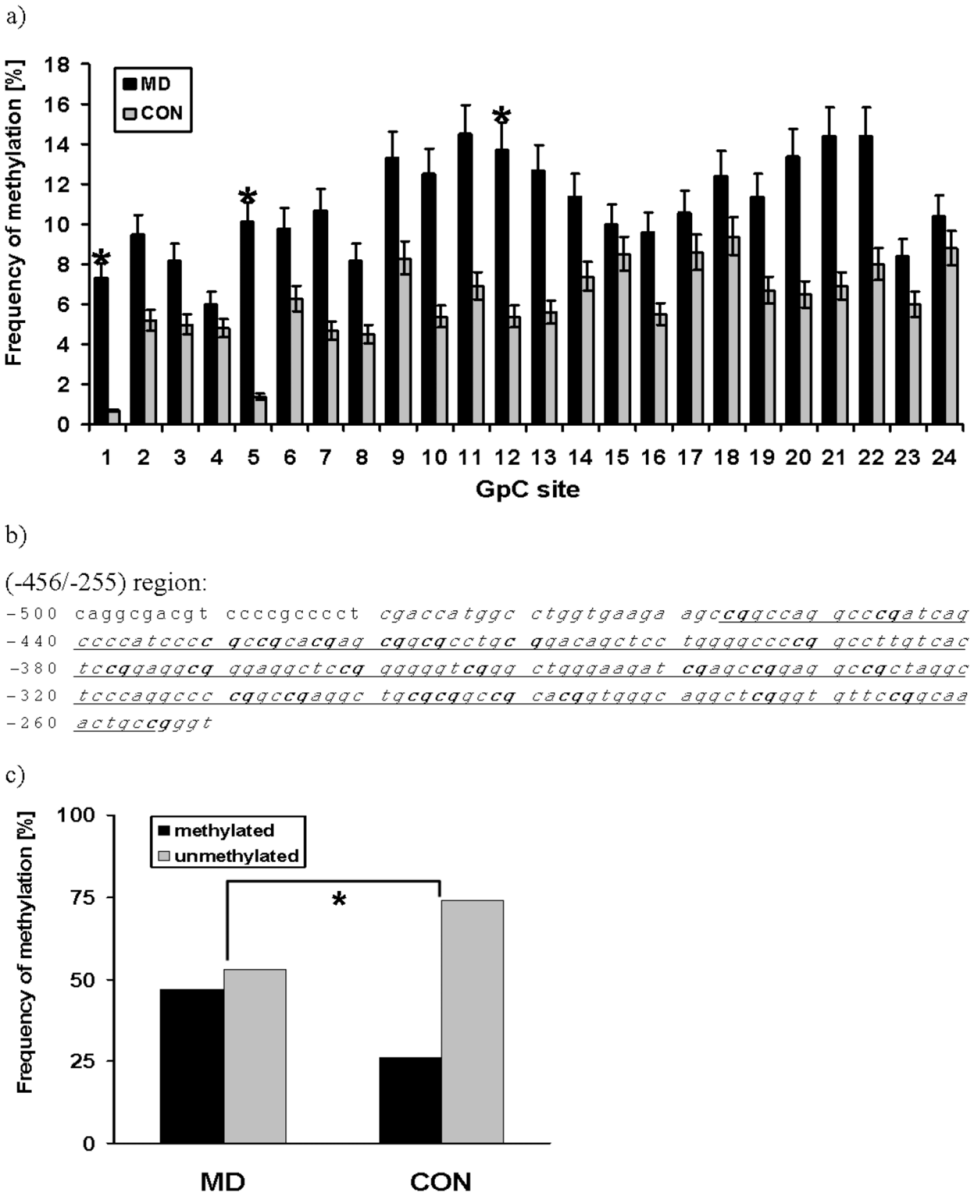
Frequency of DNA methylation in the investigated ACE promoter region. (a) Frequency of DNA methylation at 24 CpG sites, located in the (−456/−255) region of the ACE gene in depressive patients (MD) and healthy controls (CON); *: p<0.05 (χ2-test). (b) Sequence of the (−456/−255) region (italic, underlined) including the 24 investigated CpG sites (bold). (c) Frequency of methylation and non-methylation at the 24 CpG sites, located in the (−456/−255) region of the ACE gene in depressive patients (MD) and healthy controls (CON). Patients have a significantly higher methylation status than the healthy controls (p = 0.008, χ2 = 7.1, df = 1). *: p<0.05 (χ2-test).

The (−456/−255) region contains 25 CpG sites; 24 of them could be analyzed by sequencing in 81 patients and 81 controls (figure 1b). Depressive patients showed a hypermethylation pattern at all CpG sites (mean: 11.9% ±22.8% in average over all 24 CpG sites) compared to healthy controls (mean: 6.8% ±15.9%). This observation resulted in statistical significant differences at three CpG sites (site 1, 5, 12) and a trend for significance (0.05<p<0.09) at 5 CpG sites (site 7, 10, 11, 13, 21) (figure 1a). In 38 patients out of 81 (47%) methylation between one and 20 CpG sites with mean methylation frequencies between 1% and 96% (determined as mean value of the methylation frequency over all 24 CpG sites) was detectable. In the control group only 21 subjects out of 81 (26%) revealed a methylation pattern between one and 11 CpGs with lower mean methylation frequencies between 1% and 73% (over all 24 CpG sites). Figure 1c summarizes this significant difference between methylated patients and controls (p = 0.008, χ2 = 7.1, df = 1).

**Figure 2:**
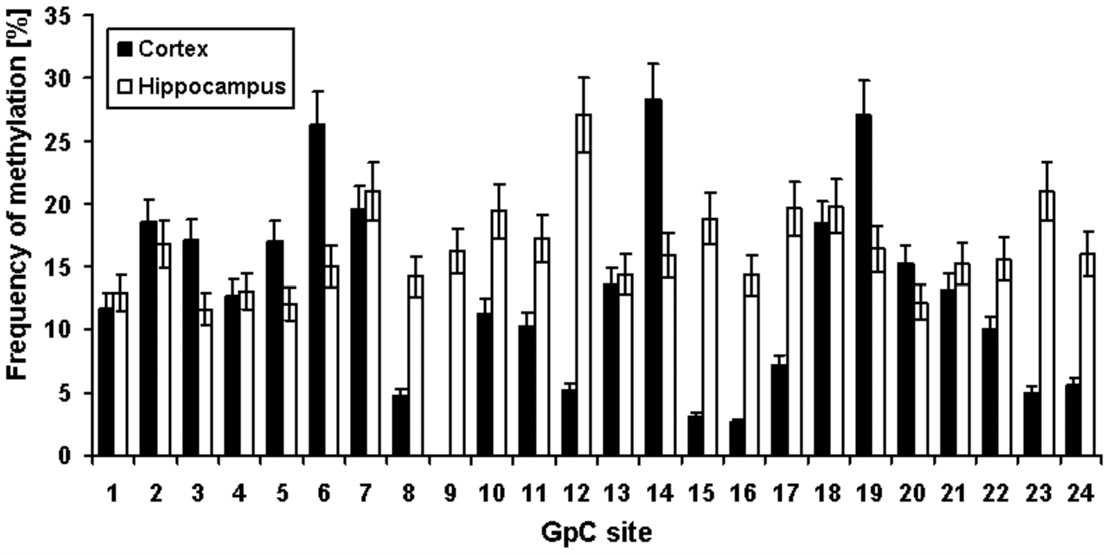
Frequency of DNA methylation at 24 CpG sites, located in the (−456/−255) region of the ACE gene in human post mortem samples of hippocampus and cortex from 13 control individuals.

Due to the partial tissue specificity of DNA methylation, we additionally analyzed the ACE promoter methylation in post mortem brain tissue to check whether the (−456/−255) ACE gene region is also a methylation “hot-spot” in brain which can be detected in neural tissues or whether the observed pattern is leukocyte specific. Data from hippocampus and cortex of 13 control individuals could clearly demonstrate a similar methylation pattern as in leukocytes of controls, but with somewhat higher frequency (figure 2). In average, methylation levels of 18% (over all 24 CpG sites) in cortex (mean: 17.8% ±19.9%) and 13% in hippocampus (mean: 12.6% ±14.0%) could be measured.

Further statistical analysis revealed no correlation between number of methylated CpG sites or methylation frequency and age, gender or post mortem interval in the analyzed patient-, control- and post mortem samples.

### Correlation between DNA Methylation of the ACE Gene and ACE Serum Concentration

**Figure 3:**
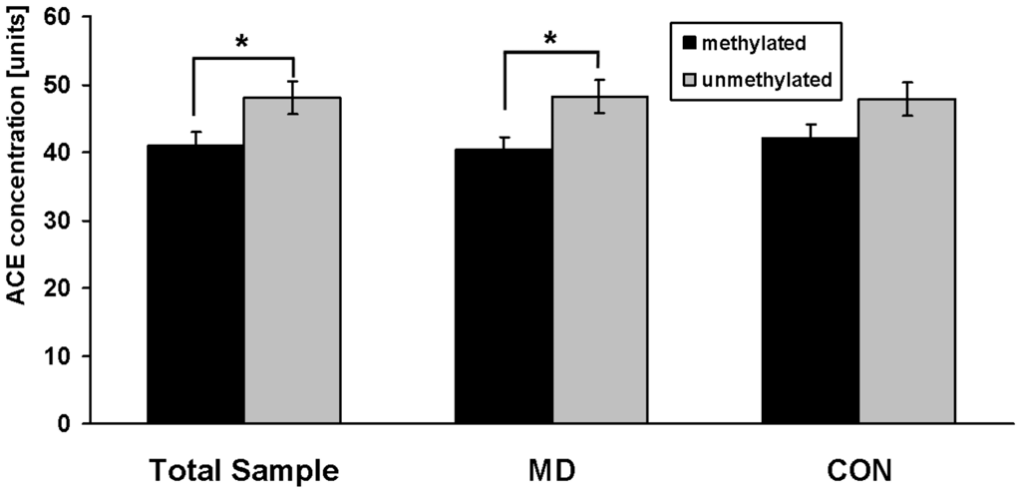
ACE serum concentrations (baseline) among the total sample, medication-free depressive patients (MD) and controls (CON) in relation to their methylation status in the (−456/−255) region of the ACE gene. Methylated probes showed lower ACE concentrations (p = 0.005, F = 8.1). This effect was also obvious in depressive patient (p = 0.03, F = 5.1). In the control group there was a similar pattern, but without statistical significance. *: p<0.05 (ANCOVA). Data are mean values ± s.e.m.

**Figure 4:**
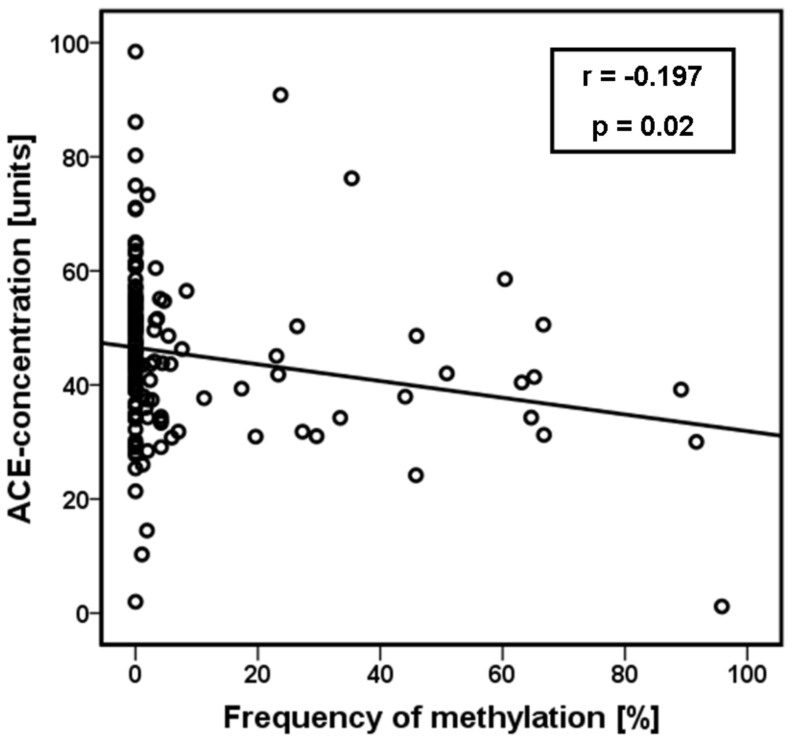
Correlation analysis between the ACE serum concentrations (baseline) and the ACE methylation frequency over all 24 CpG sites of each probe. We found a significant inverse correlation in the total sample (r = −197, p = 0.02; Pearson’s correlation).

ACE serum concentrations could be measured at baseline in 74 medication-free depressive patients and 68 controls with no differences between the groups (44.4 units ±15.4 units versus 46.2 units ±14.2 units). 36 patients and 19 controls were methylated, whereas 38 patients and 49 controls had no methylation in the investigated gene region. Analysis in groups of methylated versus unmethylated samples yielded a significant difference in the total sample in terms of lower ACE concentrations in methylated probes (p = 0.005, F = 8.1). This effect was also obvious in depressive patient, but with lower significance due the smaller sample size (p = 0.03, F = 5.1). In the control group the observed pattern was not statistically significant (figure 3). These results could be supported by correlation analysis. We found a significant inverse correlation between methylation frequency (determined as mean value of the methylation frequency over all 24 CpG sites for each probe) and the ACE serum concentration in the total sample (r = −197, p = 0.02) (figure 4). We could not identify a direct correlation between the number of methylated CpG sites and the ACE serum concentration neither in patients nor in controls. Moreover, there was no relation between age or gender and ACE levels in both groups.

### Correlation between DNA Methylation of the ACE Gene and Serum Concentration of Inflammatory CVD Markers

We found a significant positive correlation between the serum concentrations of ACE and the inflammatory CVD risk markers ICAM-1 (r = 0.26, p = 0.05), VCAM-1 (r = 0.27, p = 0.04), E-selectin (r = 0.48, p<0.001), P-selectin (r = 0.33, p = 0.02) and MCP-1 (r = 0.30, p = 0.03) in depressive patients, independent from the functional ACE Ins/Del polymorphism (data not shown). Assuming that this observation might be caused by epigenetic mechanisms, we analyzed a possible relation between the methylation pattern in the (−456/−255) ACE gene region and the inflammatory marker concentrations. Data were available from 66 medication-free depressive patients (baseline).

**Figure 5:**
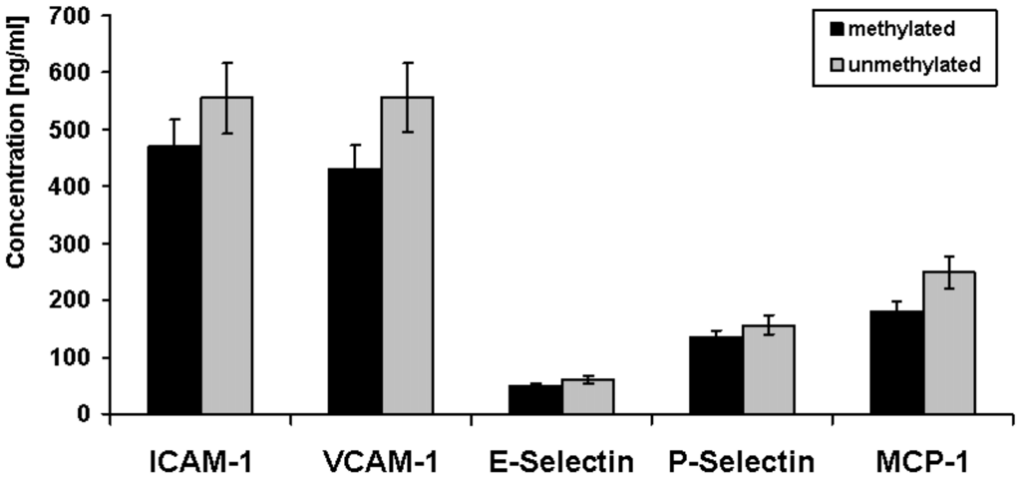
Serum concentrations of inflammatory markers (baseline) among medication-free depressive patients in relation to their methylation status in the (−456/−255) region of the ACE gene. Patients with methylated CpG sites in the (−456/−255) region showed decreased concentrations of all inflammatory markers compared to unmethylated patient samples (not statistically significant).

**Figure 6:**
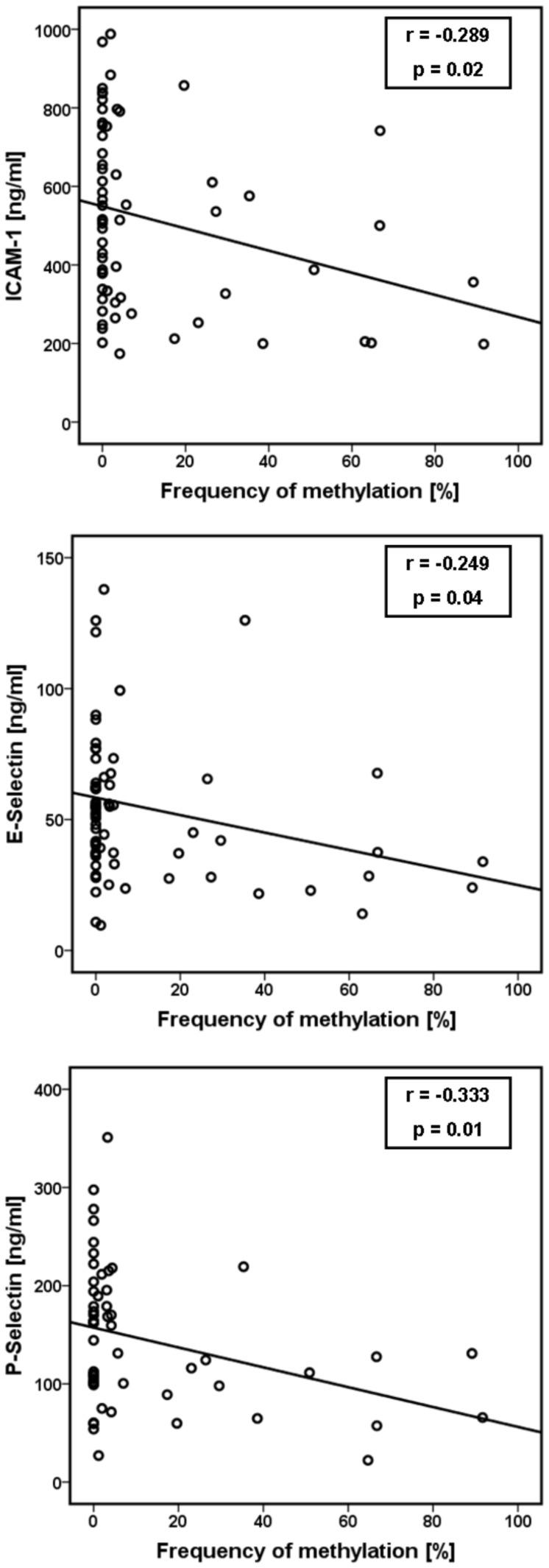
Correlation analysis between the inflammatory marker serum concentrations (baseline) and the ACE methylation frequency over all 24 CpG sites of each probe. We found a significant inverse correlation between the methylation frequency and the serum baseline concentrations of ICAM-1 (r = −0.289, p = 0.02), E-selectin (r = −0.249, p = 0.04) and P-selectin (r = −0.333, p = 0.01); (Pearson’s correlation).

As shown in figure 5 patients with methylated CpG sites in the (−456/−255) region showed only a trend for decreased concentrations of all inflammatory markers compared to unmethylated patient samples (not statistically significant). Additional correlation analyses revealed a significant inverse relation between the methylation frequency (determined as mean value of the methylation frequency over all 24 CpG sites for each probe) and the serum baseline concentrations of ICAM-1 (r = −0.289, p = 0.02), E-selectin (r = −0.249, p = 0.04) and P-selectin (r = −0.333, p = 0.01) (figure 6).

We could not identify a direct correlation between the number of methylated CpG sites and any of the inflammatory marker concentrations. Furthermore there was no relation between age or gender and the inflammatory marker concentrations.

Further analysis of patient’s methylation pattern or number of methylated CpG sites in relation to clinical variables as HAMD-17-total scores, age of onset, number of episodes, duration of clinical stay or internal parameters as weight, body mass index (BMI), blood glucose and lipids revealed no significant correlation.

## Discussion

Angiotensin-converting-enzyme (ACE) is a membrane bound endopeptidase which is involved in the metabolism of angiotensin I and bradykinin, both being important for the regulation of vascular tone and cardiac functions [28]. Besides its crucial role in cardiovascular homeostasis, the ACE gene confers also susceptibility for depression, as demonstrated in several genetic and expression studies [23], [25], [26]. Moreover the ACE gene is discussed as a common factor for the known relation between MD and CVD, but the underlying mechanisms are poorly understood [21]. Epigenetic alterations, probably via environmental influences as early stressful life events might be one explanation for these observations.

Therefore we analyzed the DNA methylation in the promoter region of the ACE gene in peripheral leukocyte DNA of 81 depressed patients and 81 healthy controls in relation to ACE serum concentrations, to inflammatory CVD risk markers and to clinical characteristics.

Bisulfite sequencing of a 1398 bp CpG island in the proximal ACE promoter revealed an intensive methylation pattern of 24 CpG sites from nucleotide −456 to −255 with significantly higher methylation levels in depressive patients versus controls. DNA methylation within this (−456/−255) region could also be demonstrated in post mortem hippocampus and cortex tissue of 13 control individuals. Taken together, these findings demonstrate that in almost 50% of depressed patients promoter methylation of the ACE gene is detectable with a two-fold higher frequency as in controls. Our data further demonstrate that a comparable methylation pattern as in peripheral lymphocytes is present in cortex and hippocampus, which suggest also an important role of ACE promoter methylation in the brain.

The anterior cingulate cortex, amygdala and hippocampus form part of an interconnected prefrontal-limbic network (PLN) that is dysregulated in depressive disorders [29]. The PLN is modulated by the basal ganglia and midbrain structures, as well as by the HPA-axis. The ACE gene is not only expressed in the PLN [30], [31], but also involved in HPA-axis dysregulation in depressed patients, as we have reported in previous studies [22], [25]. Moreover, epigenetic alterations within the PLN in relation to early life stress, a known risk factor for depression, have been reported for the glucocorticoid receptor gene in animal studies [32]. Therefore it seems probable that epigenetic alterations of the ACE gene in terms of hypermethylation might contribute to the dysregulated PLN in depression, at least in a subgroup of patients. We are aware that these conclusions from our data are highly speculative because only post mortem brain samples of controls, which died mostly from cardiovascular diseases, were included in the analysis due to the lack of brain tissue from MD patients. Thus it is not possible to draw meaningful conclusions.

Interestingly our findings of intensive DNA methylation in the (−456/−255) ACE gene region are consistent with a recent report by Rivière et al. (2011), who investigated the influence of DNA methylation and chromatin condensation state on the expression of the human ACE gene in human liver (HepG2), colon (HT-29), microvascular endothelial (HMEC-1) and lung (SUT) cell lines [33]. Within a 3340 bp fragment of the putative ACE promoter region, they described two CpG island (−562/−244 and −202/216) after in silico analysis, which are both located in the CpG island, identified in our study. Similarly to our results they found almost exclusively intensive DNA methylation at the 25 CpG sites in the (−456/−255) region, with highest levels in HepG2 cells followed by HMEC-1, HT-29 and SUT cells. This proximal region contains several transcription factor response elements as EGR-1, SP 1, CP 2 and was also reported in an earlier study to be sufficient to drive high transcriptional activity [34]. Furthermore, within luciferase reporter gene assays Rivière et al. (2011) could demonstrate that a 3340 bp fragment of the 5′-ACE region which includes the (−456/−255) fragment induces cell type specific promoter activity which is dependent of the methylation level. Additionally, the methylation pattern was clearly related to the ACE mRNA concentrations in vitro as well as in vivo (rats) [33]. These observations confirms that the (−456/−255) region within the promoter of the ACE gene is of outstanding functional importance for ACE expression.

To analyze a probable relation between DNA methylation pattern and ACE expression, ACE serum concentrations from 74 medication-free depressive patients at baseline and 68 controls were available. Although there were no significant differences in ACE serum concentrations between patients and controls, methylated probes in the (−456/−255) region had significantly lower ACE concentrations than unmethylated ones in the total sample. Moreover, this observation was supported by an inverse correlation between the ACE serum concentration and the methylation frequency, which suggest that ACE promoter methylation affects serum ACE protein levels. With regard to the significant higher ACE promoter methylation frequency in depressed patients it seems probable that these epigenetic mechanisms have a higher impact on depression than on healthy controls.

In contrast to the results from Rivière et al. (2011), who reported a significant relation between the number of methylated CpG sites in the (−456/−255) region and the ACE mRNA expression in HMEC-1, HT-29 and HepG2 cells, we could not detect any significant direct correlation between methylated CpG site number and ACE protein concentrations in serum [33]. This is not surprising, particularly if one takes into account that the protein expression is not exclusively dependent on the gene transcription level, but also on transport mechanisms, mRNA localisation, transcriptional stability, regulation of translation and post translational modifications [35].

Besides the brain specific functions of the RAS, as brain development, learning and memory, sympathetic activation, vasopressin release and regulation of the stress response, its classical role is described as a circulating hormone system focused on cardiovascular regulation and body fluid homeostasis with angiotensin II (Ang II) as its main effector [36], [37]. MD represents a major risk factor for cardiovascular disease (CVD) independently of traditional risk factors and on the other hand CVD influences affective states and influence depression [38], [39]. Thus, due to this bi-directional relationship the RAS was intensively investigated during the last few years as common pathological mechanism for both disorders. In this context, functional polymorphisms of the ACE gene have been linked to depression, hypertension and myocardial infarction [25], [40]–[42]. In addition, CVD risk marker level, as C-reactive protein (CRP), HPA axis abnormality and the speed of response during antidepressant treatments were related to ACE gene polymorphisms [43] [22]–[24]. These findings clearly indicate the central role of the ACE gene as candidate gene for both disorders and for their bi-directional relationship, but the underlying molecular mechanisms are poorly understood. Thus, epigenetic regulations of the ACE gene become more and more a major research topic in the physiological role of ACE and future studies have to clarify whether epigenetics might superimpose or be totally independent from functional genetic variants, such as the Ins/Del polymorphism [44].

We further investigated to what extent MD is related to inflammatory CVD markers as ICAM-1, VCAM-1, E-selectin, P-selectin and MCP-1 in cardiovascularly healthy patients suffering from MD [45]. Besides an increase of many inflammatory markers in MD patients, we found a significant correlation between the ACE serum concentrations and several of these markers in these patients, independent from the functional ACE Ins/Del polymorphism (unpublished data). Due to this observation we assumed that epigenetic alterations of the ACE gene might cause this effect. Therefore we evaluated a possible correlation between the methylation pattern in the (−456/−255) ACE gene region and the inflammatory marker concentrations in sera of 66 medication-free depressive patients at baseline.

The results seem to partially confirm our hypothesis. We observed a trend for decreased concentrations of all inflammatory markers in depressed patients with methylated CpG sites in the (−456/−255) region. Correlation analysis revealed a significant inverse relation between the ACE promoter methylation frequency and the serum concentrations of ICAM-1, E-selectin and P-selectin in depressed patients.

Numerous animal and human studies could demonstrate a relation between ACE and inflammatory CVD markers in terms of a reduction of these markers caused by ACE inhibitor treatment which affects the Ang II generation [46]–[48]. Moreover, a previous report have shown reduced renal ACE mRNA concentrations in rats after treatment with the ACE inhibitor lisinopril [49]. In this context it might be possible that, besides an inhibition of ACE also lower ACE concentrations itsself caused by higher rates of ACE methylation, have a protective effect on the development of CVD. To our knowledge, the results of the present study demonstrate for the first time that differential DNA methylation patterns in the promoter region of the ACE gene seem to affect the expression of inflammatory CVD risk marker concentrations in depression. This interaction might be involved in the pathophysiological mechanisms which predispose a subgroup of depressed patients to develop CVD or not. This could be one of the missing links of the bi-directional relation between MD and CVD.

We are aware that several limitations should be considered when interpreting our findings and that our conclusions are partially very speculative at the moment. First, the sizes of patient and control samples are probably not large enough for final conclusions. This might also be a reason for the lack of a significant relation between clinical variables like HAMD-17-total scores, treatment response, age of onset, number of episodes or duration of clinical stay and the methylation frequency in the ACE promoter.

Second, the use of peripheral leukocyte DNA limits the inference that can be drawn regarding pathways involving the brain. Nevertheless, a growing body of research confirms the promise of using blood cells as peripheral model [50], [51]. Human and animal studies in neurological disorders such as stroke, multiple sclerosis or autism using microarrays could clearly demonstrate specific disease related gene profiles in blood of patients including many brain specific genes [52]. Moreover, numerous epigenetic studies of psychiatric disorders have been performed in peripheral leukocyte DNA [53], and there is evidence suggesting a DNA methylation concordance between peripheral tissues and brain for genes like catechyl-o-methyltransferase (COMT) [54], [55]. Only a limited number of studies have investigated common central and peripheral ACE alterations. For example, Tan et al. (2004) measured increases in brain and cardiac ACE densities after myocardial infarct in rats [56].

In the present study we compared brain and blood ACE promoter methylation in controls using post mortem brain tissues of hippocampus and frontal cortex. Total methylation levels of 18% in cortex and 13% in hippocampus could be measured compared to ∼7% in peripheral leukocytes of controls. Although the post mortem samples came from individuals who died mostly from cardiovascular diseases which hampers any conclusions regarding differences in methylation frequencies between blood and brain controls, it appears that the (−456/−255) ACE gene region is also a probable “hot-spot” for methylation in these brain regions. On the other hand the differences in methylation frequencies between post mortem and blood samples might be a result of tissue specificity of ACE regulation, suggesting less ACE gene expression in cortex and hippocampus than in peripheral leukocytes. Therefore, it remains the question how brain and peripheral ACE features parallel to each other. From the present data we cannot answer this question in full detail, but we hypothesize that the observed methylation pattern in peripheral blood cells might represent a disease specific profile. Further studies in different brain tissues of depressed patients and healthy controls are needed to validate this hypothesis.

Moreover, the peripheral DNA samples are a mix of mononuclear cells, which derive from two distinct lineages and compartments. Since cell count information was not available, we cannot completely rule out whether the differences in methylation frequencies reflect different relative numbers of mononuclear cells in the cases versus the controls.

Further, our data represent correlative findings and thus cannot prove a causal relationship between ACE DNA methylation – ACE mRNA expression – ACE protein expression, respectively inflammatory CVD marker expression, because we had no mRNA from patients and controls of the present study. It has also to be considered that soluble serum ACE originates from endothelial cells, mainly from lung shed from the cell membrane by proteolytic cleavage, but at physiologic conditions the concentration of ACE in blood is very stable and thus serum ACE measurements are an essential tool for monitoring the level, respectively activity of ACE [57], [58].

Furthermore, DNA methylation represents a mixture of state- and trait dependent effects. Further prospective studies have to clarify whether the observed DNA methylation revert to control levels after remission, respectively whether there are similar abnormalities in first degree relatives of the patients.

Otherwise, despite these limitations, the functional significance of our data is widely supported by the recent distinguished work of Rivière et al. (2011) who could clearly demonstrate the functional significance of DNA methylation in the (−456/−255) ACE gene region for promoter activity as well as for mRNA expression in vitro and in vivo [33]. Thus, we believe that the limitations of our study do not detract from the major significance of its results, which will need further confirmation in independent studies.

In summary, this study is the first to demonstrate that the human ACE expression is under a strong epigenetic influence by DNA methylation in MD. Approximately 50% of depressed patients of the present study showed DNA methylation in a functional relevant 201 bp region of the ACE gene proximal promoter in peripheral leukocytes compared to only ∼25% of healthy controls. This methylation pattern seems to have an influence on serum ACE protein expression in the total sample and additionally on the amount of inflammatory risk markers for CVD, as ICAM-1, E-selectin and P-selectin in depressed patients. Although several questions remain to be resolved regarding the precise functional consequences of the identified ACE promoter DNA methylation, our data are consistent with the hypothesis that DNA methylation aberrations may be an underlying cause of depressive disorders. Furthermore, the present findings support the importance of CpG methylation of the ACE gene as a common pathogenic factor in MD and CVD and may lead to a better understanding of the neurobiology of MD, as well as of the bi-directional relationship between MD and CVD.

## Materials and Methods

### Ethics Statement

All clinical investigations have been conducted according to the principles expressed in the Declaration of Helsinki and approved by the Ethics Committee of the Medical Faculty of the Ludwigs Maximilians University (LMU) Munich (Head: Prof. Dr. Wolfgang Eisenmenger, Members: Prof. Dr. Eckhard Held, Prof. Dr. Gustav Paumgartner, PD Dr. Thomas Beinert, Prof. Dr. Hans Ulrich Gallwas, Prof. Dr. Detlef Kunze, Dr. Viktoria Mönch, Prof. Dr. Randolph Penning, Prof. Dr. Klaus Hahn, Prof. Dr. Klaus Jürgen Pfeifer, and Dr. Christian Zach). Ethics proposal, Project No. 213/00; positive vote from: 12.05.2005 “Genetische, biochemische und funktionelle Untersuchungen an depressiven Patienten und gesunden Kontrollpersonen”. Written informed consent was given by the patients and healthy volunteers. Autopsy samples: The autopsies were court ordered from the state attorney according to the German legal situation due to unknown causes of death. In that case informed consent from the next of kin is not required, because relatives have no possibility for intervention. Within these autopsies it is necessary to take routinely additional tissue probes for probable further investigations. The probes of the present study originate from these investigations. The Ethics Committee of the LMU Munich approved this procedure. All autopsies were performed according to the legal requirements. For the control individuals the natural cause of death was verified finally by these autopsies. Blood and brain samples were exclusively taken during the routine autopsies to perform the court ordered analysis. Furthermore post mortem material will be preserved for subsequently necessary investigations on behalf of the state attorney. Blood and brain samples were never taken for research. For research projects we use only remaining post mortem samples which have been released and approved for use in research by the Ethics Committee of the LMU Munich. As described, the consent for research use of autopsy tissues will be given by the local Ethics Committees of the universities. This is the current procedure in legal medicine in Germany.

### Subjects

Depressed patients and healthy controls were taken from two cohorts recently published in an interdisciplinary study by Baghai et al. (2010) [45]. In brief, 81 unrelated Caucasian patients suffering from unipolar major depression (30 males, 51 females, mean age 45.8 years ±14.3 years) were recruited from in-patients at the Department of Psychiatry and Psychotherapy of the Ludwig-Maximilian-University of Munich (LMU). Patients were diagnosed by experienced and trained psychiatrists according to DSM-IV using the Structured Clinical Interview for DSM-IV disorders (SCID-I) [59]. The main inclusion criteria were unipolar depression and a score in the Hamilton Rating Scale for Depression (HAM-D17) of at least 17. Prior to inclusion in the study blood samples were obtained for routine laboratory screening, a medical history was taken and a physical examination was performed by a physician to exclude severe medical disorders. Clinically relevant medical illness and the concomitant use of antihypertensive medications such as ACE inhibitors or angiotensin receptor blockers as well as beta blockers and hormone replacement therapies, alcohol or drug abuse within the last 6 month prior to study inclusion or withdrawal signs led to exclusion from the study. Blood samples were taken after a washout period of at least 3 days.

81 ethnically matched subjects from the general population (40 males, 41 females, mean age 46.2 years ±14.2 years) served as control group. All subjects were psychiatrically screened by a short structured interview with a psychiatrist to rule out psychiatric problems. Subjects with known history of psychiatric disorders were excluded from the study.

All patients and controls were of Caucasian origin from the German population and came from the same geographical area in southern Germany. Demographic data of patients and controls are given in table 1.

### Post Mortem Samples

Brain specimens derived from 13 individuals (7 males, 6 females, mean age 41.9 years ±16.3 years) who died suddenly from diseases not directly involving the CNS were obtained from the Institute for Legal Medicine of the LMU. Causes of death were the following: acute cardiac failure (n = 4), accident (n = 4), aortic aneurysm (n = 2) and homicide (n = 3). The clinical, respectively medical data sheets of the individuals were available to the Institute for Legal Medicine of the LMU and did not give any hint on lifetime psychiatric or neurological disorders. Accordingly to the medical records there was no history of psychopharmacological medication, alcohol or drug abuse. All individuals were Caucasian from the same geographical region in southern Germany.

The unfixed brain specimens were obtained 4–29 hours (average post mortem delay of 15.8 hours ±7.3 hours) after death during routine autopsy. Sections were taken from the posterior hippocampus at the coronal level of the lateral geniculate nucleus and from the cerebellar cortex. The tissue probes were collected using the RNAlater kit (Qiagen, Hilden, Germany) and immediately frozen at −80 C until used for the DNA extraction.

### Promoter CpG Island Analysis

We selected a 7540 bp region (−5989/+1551, related to the transcription start site), containing the putative promoter and exon 1 of the ACE gene (NG_011648: nt 1 to 6540). CpG content analysis was performed applying the CpG Island Searcher (http://www. uscnorris.com/cpgislands2/cpg.aspx) [60]. The CpG islands were defined as a region with at least 500 bp, with a GC percentage that is greater than 55% and with an observed/expected CpG ratio that is greater than 65%.

### Bisulfite Sequencing of Genomic DNA

Genomic DNA was extracted from whole blood of patients and controls, as well as from the post mortem brain samples with the Invisorb Blood Giga Kit (Invitek, Berlin, Germany) according to the manufacturer’s instructions. The peripheral DNA samples originated from several genetic projects. Therefore, cell counts to differentiate between the different mononuclear blood cells were not available.

750 ng of genomic DNA were treated with sodium bisulfite using the EpiTect Bisulfite Kit (Qiagen, Hilden, Germany) according to the manufacturer’s instructions.

Five primer sets were designed to amplify the CpG island region in overlapping fragments. Given that we exclusively found high degree of DNA methylation in the PCR fragment of primer set 2, the following method description refers only to primer set 2. Detailed information about the further PCR sets, as well as the PCR and sequencing conditions can be obtained on request.

Both primers harboured universal primer sequences at their 5′-end to facilitate sequencing. The following sequences were designed for primer set 2: forward primer (NG_011648: −465/−447, related to the transcription start site): 5′-M13(-21)-TTA TGG TTT GGT GAA GAA GT-3′; reverse primer: (NG_011648: −231/−210, related to the transcription start site): 5′-M13(-29)-AAA AAA ACC TCC TCT CTT TAA A-3′.

PCR was carried out in a final volume of 10 µl containing 1 µl of bisulfite treated DNA, 200 µM of each dNTP, 5 mM MgCl_2_, 2 µl Q-Solution, 0.5 µM of the forward and reverse primer and 0.5 units Taq DNA polymerase (Qiagen, Hilden Germany). After an initial denaturation step of 95°C for 15 min, there was a first round of 5 cycles of denaturation at 95°C for 30 sec, annealing at 58°C for 90 sec and extension at 72°C for 120 sec. The second PCR round consisted of 35 cycles of denaturation at 95°C for 30 sec, annealing at 54°C for 90 sec and extension at 72°C for 90 sec. A final step was performed at 72°C for 5 min. PCR products were purified by SAP (shrimp alkaline phosphatase) treatment according to standard protocols (Applied Biosystems, Foster City, CA, USA).

50 ng of the purified PCR product was used for cycle sequencing with the BigDye Terminator 3.1 Cycle Sequencing Kit (Applied Biosystems, Foster City, CA, USA) in a final volume of 20 µl containing 3 µl reaction mix and 0.16 µM pimer. Results were verified by bi-directional sequencing. Cycling conditions were: 35 cycles of denaturation at 96°C for 10 sec, annealing at 50°C for 5 sec and extension at 50°C for 4 min. The amplified products were ethanol/EDTA purified according to the described conditions by Applied Biosystems. Samples were sequenced on an ABI 310 capillary sequencer (Applied Biosystems, Foster City, CA, USA). Data were processed by using Sequencing Analysis version 5.3.1 applying the KB-Base Caller 1.3 (Applied Biosystems, Foster City, CA, USA).

The approximate methylation frequency of cytosine of each CpG site was calculated by comparing the peak height of the cytosine signal with the sum of the cytosine and thymidine peak height signals, as described by Melki et al. (1999) [61]. This method was validated by Jiang et al. (2010) [62], who could confirm after comparison with pyrosequencing and bisulfite-clonig sequencing that this method is a simple, high-throughput and a reliable technology for determining the methylation status of specific genes. However, when using direct sequencing, it is often difficult to assess methylation levels <15% due to variable sequencing background signal. Thus, CpG sites with ratio ranges 0.00–0.20 were considered as unmethylated (0%) and ranges between 0.81–1.0 as fully methylated (100%). For the ratio range 0.21–0.80 the calculated frequencies were used.

### Measurement of ACE Concentration

Blood samples for ACE determination were drawn from medication-free MD patients and controls before the first application of antidepressant treatment (baseline). Aprotenin treated serum was stored frozen at −80°C until assessment. ACE was quantified using a commercial radio enzyme assay (REA) (ACE-REA, Diagnostic Products Corporation Biermann, Bad Nauheim, Germany). The lower detection limit was 3.8 U/l.

### Measurement of Inflammatory Markers

The inflammatory markers were analyzed in serum of medication-free patients before pharmacological treatment (baseline). MCP-1, VCAM-1, ICAM-1, E-selectin, and P-selectin, were determined using ELISAs obtained from IBL (R&D Systems, Minneapolis, USA) according to manufacturer’s instruction.

All laboratory analyses were carried out blind to case control status.

### Statistical Analysis

All statistical analyses were performed with SPSS for Windows (Version 19.0; SPSS; Chicago, IL).

All dependent variables (concentrations of ACE and inflammatory markers) were examined by the Kolmogorov-Smirnov test on normality. Stepwise linear regression analyses (pin  = 0.05, pout  = 0.10) with the independent variables gender and age were performed for all dependent variables. Correlations analyses between dependent variables and age, as well as methylation pattern and between methylation pattern and post mortem interval (PMI) in post mortem samples were performed with the Pearson’s test.

Analyses of covariance (ANCOVA) were conducted to test group differences with regard to DNA methylation pattern with age and gender as covariates. The level of significance was set at 0.05.
